# Characterization of an autotransporter adhesin protein shared by *Burkholderia mallei* and *Burkholderia pseudomallei*

**DOI:** 10.1186/1471-2180-14-92

**Published:** 2014-04-14

**Authors:** Eric R Lafontaine, Rachel Balder, Frank Michel, Robert J Hogan

**Affiliations:** 1 Department of Infectious Diseases, University of Georgia College of Veterinary Medicine, 30602 Athens, GA, USA; 2 Department of Veterinary Biosciences and Diagnostic Imaging, University of Georgia College of Veterinary Medicine, 30602 Athens, GA, USA

## Abstract

**Background:**

Autotransporters form a large family of outer membrane proteins specifying diverse biological traits of Gram-negative bacteria. In this study, we report the identification and characterization of a novel autotransporter gene product of *Burkholderia mallei* (locus tag BMA1027 in strain ATCC 23344).

**Results:**

Database searches identified the gene in at least seven *B. mallei* isolates and the encoded proteins were found to be 84% identical. Inactivation of the gene encoding the autotransporter in the genome of strain ATCC 23344 substantially reduces adherence to monolayers of HEp-2 laryngeal cells and A549 type II pneumocytes, as well as to cultures of normal human bronchial epithelium (NHBE). Consistent with these findings, expression of the autotransporter on the surface of recombinant *E. coli* bacteria increases adherence to these cell types by 5–7 fold. The gene specifying the autotransporter was identified in the genome of 29 *B. pseudomallei* isolates and disruption of the gene in strain DD503 reduced adherence to NHBE cultures by 61%. Unlike *B. mallei*, the mutation did not impair binding of *B. pseudomallei* to A549 or HEp-2 cells. Analysis of sera from mice infected via the aerosol route with *B. mallei* and *B. pseudomallei* revealed that animals inoculated with as few as 10 organisms produce antibodies against the autotransporter, therefore indicating expression in vivo.

**Conclusions:**

Our data demonstrate that we have identified an autotransporter protein common to the pathogenic species *B. mallei* and *B. pseudomallei* which mediates adherence to respiratory epithelial cells and is expressed in vivo during the course of aerosol infection.

## Background

Autotransporter proteins are the largest known family of virulence factors expressed by Gram-negative bacteria and play prominent roles in processes such as invasion [1], serum resistance [2,3], phospholipolysis [4-6], cytotoxicity [7], adherence [8,9], survival within eukaryotic cells [10], intracellular motility [11], cell-to-cell aggregation [12,13], and biofilm formation [14,15]. These molecules display conserved structural features including an N-terminal surface-exposed domain responsible for the biological function and a hydrophobic C-terminus that tethers the autotransporter to the outer membrane (OM). Based on the structure of the C-terminus, autotransporters can be classified as conventional or oligomeric [16-21]. The C-terminus of conventional autotransporters consists of ~300 amino acids (aa) forming 10–12 antiparallel β-strands, while that of oligomeric autotransporters is substantially shorter (~70 aa) and specifies only 4 β-strands. Because of their structure and role in virulence, autotransporters are attractive targets for developing countermeasures against pathogenic organisms. Large portions of autotransporters are located on the bacterial surface and therefore readily accessible for recognition by the immune system. Additionally, autotransporters play important roles in pathogenesis, thus targeting them may hinder the ability to cause disease. This hypothesis is supported by several studies demonstrating the effectiveness of autotransporter-based countermeasures. For example, immunization with *Neisseria meningitidis* NadA elicits antibodies (Abs) binding to the bacterial surface and promoting complement-mediated killing [22,23], which is key to protection against this organism. Antibodies against *Haemophilus influenzae* Hap block adherence to epithelial cells and immunization with Hap protects mice in nasopharyngeal colonization studies [24,25]. Vaccination with the *Proteus mirabilis* autotransporter cytotoxin Pta yields Abs that not only reduce bacterial burden in a murine urinary tract infection model, but also neutralize the cytotoxic activity of Pta for bladder cells [26]. Moreover, *Bordetella pertussis* Pertactin, an autotransporter adhesin, is a component of licensed vaccines for whooping cough (http://www.cdc.gov/vaccines/pubs/pinkbook/downloads/pert.pdf).

*Burkholderia mallei* and *Burkholderia pseudomallei* are closely related Gram-negative organisms for which developing efficacious countermeasures is highly desirable. Both species are classified as Tier 1 agents by the U.S. Federal Select Agent Program because of concerns regarding their use as bioweapons, especially since *B. mallei* has been utilized in this manner on more than one occasion [27-31]. *Burkholderia mallei* is a host-adapted pleomorphic coccobacillus that does not persist in the environment outside of its natural equine reservoir. The bacterium causes the highly contagious zoonotic disease glanders, which primarily affects horses, and is endemic to parts of Asia, Africa, South America and the Middle East [27,32-38]. In humans, infection typically occurs via the cutaneous or aerosol route upon contact with infected animals. Clinical manifestations include fever, pneumonia, necrosis of the trachea and bronchi, bacteremia, and dissemination of *B. mallei* to organs where it causes necrotizing abscesses. *Burkholderia pseudomallei* is a saprophyte of wet soils and is endemic to countries bordering the equator. The organism can infect most mammals and causes the disease melioidosis in humans, a febrile illness that varies greatly in its clinical presentation. Disease states range from flu-like malaise to septicemia, chronic abscess formation in deep tissues, or bacteremic pneumonia [33,39-45]. Infection is generally acquired by percutaneous inoculation, ingestion and inhalation of aerosols, and the risk of contracting disease is proportionate to the concentration of *B. pseudomallei* in soil. *Burkholderia pseudomallei* is a leading cause of sepsis and bacteremic pneumonia in Southeast Asia and Australia, and melioidosis is increasingly recognized as an emerging infectious diseases in many tropical regions of the world [40,46,47].

Glanders and melioidosis have high mortality rates (up to 50%) despite aggressive antimicrobial therapy. The recommended treatment involves the use of ceftazidime and meropenem (intensive phase) and TMP-SMX and co-amoxiclav (eradication phase) for several months [48]. Response to treatment is slow and eradication of the agents is difficult, often resulting in protracted alternating bouts of remission and exacerbation. There are no vaccines available to protect against either *Burkholderia* species. Clearly, there is a need to identify and characterize targets for developing countermeasures for these organisms. The genomes of *B. mallei* and *B. pseudomallei* have been reported to encode multiple autotransporters [49-51]. In this study, we examined one of these gene products and evaluated it role in adherence in vitro and virulence in a mouse aerosol model of infection.

## Results

### Identification of a gene encoding a potential autotransporter adhesin shared by *B. mallei* and *B. pseudomallei*

**Figure 1:**
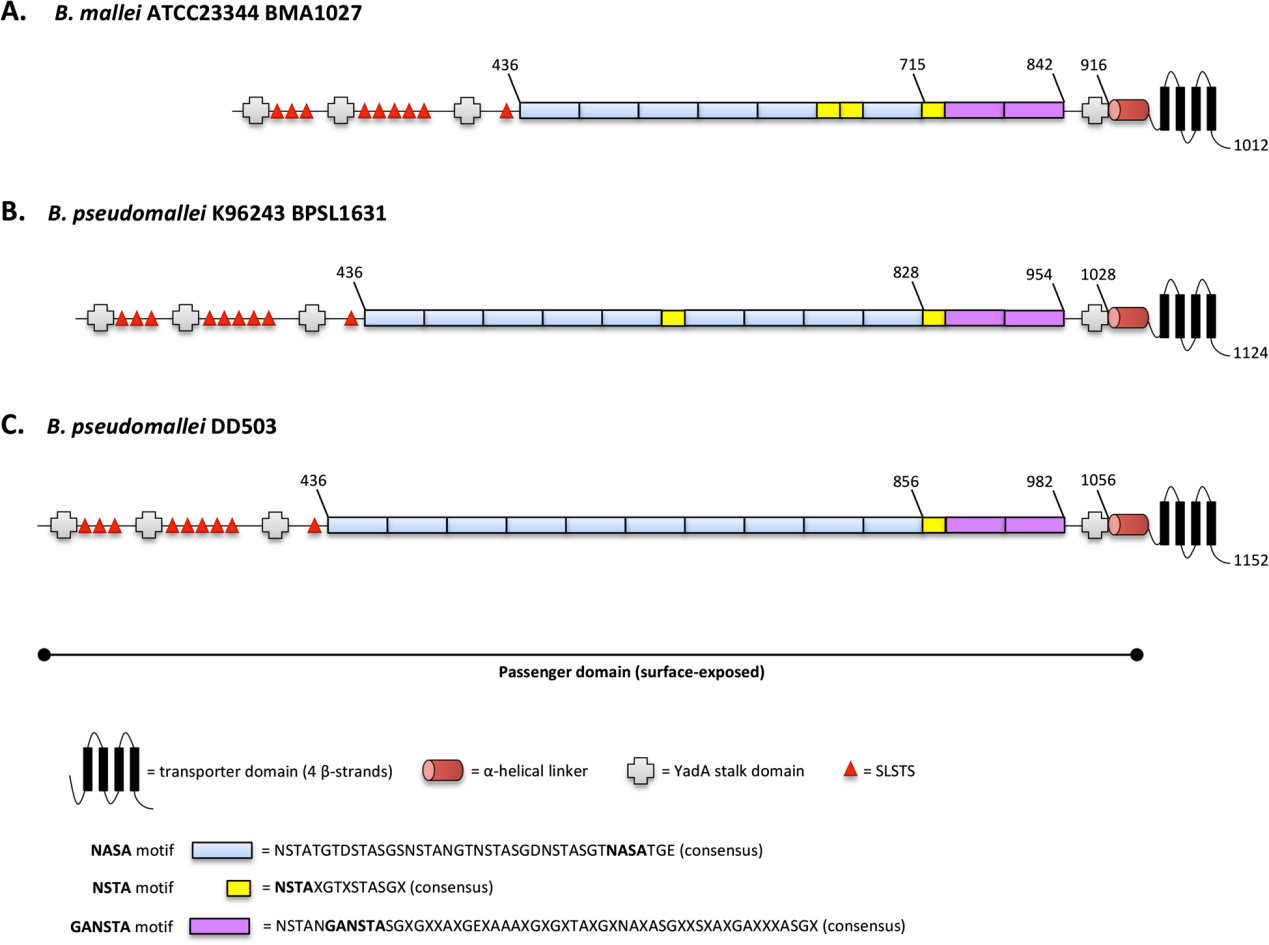
**Structural features of BMA1027 and orthologous gene products.** Different regions of the protein encoded by *B. mallei* ATCC 23344 BMA1027 **(A)**, *B. pseudomallei* K96243 BPSL1631 **(B)** and the *B. pseudomallei* DD503 BMA1027 ortholog **(C)** are depicted with the positions of residues defining selected domains. Transporter domains (OM anchors) and helical linkers were identified using the PSIPRED secondary structure prediction algorithm. The colored boxes, red triangles, and grey crosses show the relative position and number of repeated aa motifs.

Comparative sequence analyses identified a gene product in the published genome of *B. mallei* strain ATCC 23344 (locus tag # BMA1027) that resembles the adhesins *Yersinia enterocolitica* YadA [2,21,52], *Moraxella catarrhalis* Hag [8,53,54], *B. pseudomallei* BoaA and BoaB [55], and *B. mallei* BoaA [55]. These molecules belong to the **o**ligomeric **c**oiled-coil **a**dhesin (Oca) sub-family of oligomeric autotransporter proteins and have a characteristic modular organization consisting of: *(i)* a surface-exposed region specifying adhesive properties termed passenger domain, *(ii)* a short linker region predicted to form an α helix, and *(iii)* a hydrophobic C-terminus composed of four β-strands anchoring the autotransporter in the OM designated transporter domain [16,19-21]. As shown in Figure 1A, BMA1027 is predicted to possess these structural features.

Searches using the Pfam database revealed that the region encompassing aa 936–1012 of BMA1027 shows similarity to a YadA anchor domain (PF3895.10; expect value of 6.3e^−22^), which is present in most Oca and described as important for oligomerization and targeting autotransporters to the OM. Pfam searches also indicated that BMA1027 contains four YadA stalk domains (PF05662, formerly designated HIM; expect values ranging from 2.2e^−4^ to 1.5e^−9^; grey crosses in Figure 1A). This motif is associated with invasins and haemagglutinins and is present in YadA as well as Hag [2,8,52,53]. YadA contains one stalk domain, which has been shown to be necessary for protein stability and adhesive properties. Further sequence analysis revealed that the passenger domain of BMA1027 specifies repeated aa motifs, a trait noted in several oligomeric autotransporters including YadA [2,52], Hag [8,53], BoaA and BoaB [55], the *B. pseudomallei* biofilm factor BbfA [56], and the *M. catarrhalis* UspA1, UspA2, and UspA2H proteins [57-60]. As illustrated in Figure 1A, the passenger domain of BMA1027 contains nine copies of the 5-mer SLSTS (red triangles) and several repeated elements beginning with residues NSTA (colored boxes). Additional characteristics of the predicted protein are listed in Table 1.

**Table 1:** **Characteristics**^**a **^**of BMA1027 orthologous genes and their encoded products**

**Strain**^**b**^	**Locus tag**	**Predicted protein (aa)**	**MW (kDa)**	**Potential signal sequence cleavage site**^**c**^
***B. pseudomallei***				
1026b/DD503*	BP1026B_I1575	1,152	107.4	ASA^37▼^G, AMA^69▼^A
K96243	BPSL1631	1,124	104.8	ASA^37▼^G, AMA^69▼^A
***B. mallei***				
ATCC 23344	BMA1027	1,012	94.7	ASA^37▼^G, AMA^69▼^A

^a^Sequence analyses were performed using Vector NTI (Life Technologies™) and online tools available through the ExPASy Bioinformatics Resources Portal.

^b^Genomic sequences are available through the NCBI genomic BLAST service.

^c^The putative signal sequence cleavage sites were determined using the SignalP 4.1 server.

^*^The *B. pseudomallei* strain DD503 is a derivative of isolate 1026b in which the AmrAB-OprA antibiotic efflux pump has been deleted to facilitate mutant construction [61]. The BMA1027 orthologs of strains DD503 and 1026b are identical (confirmed by nucleotide sequence analysis, data not shown).

The published genomic sequence of the *B. pseudomallei* strain K96243 was found to specify a BMA1027 ortholog (locus tag # BPSL1631, Figure 1B) that is 89% identical to that of *B. mallei* ATCC 23344. The BMA1027 ortholog was sequenced from the *B. pseudomallei* strain used in our laboratory, DD503, and was predicted to encode a protein that is 97% and 87% identical to that of *B. pseudomallei* K96243 and *B. mallei* ATCC 23344, respectively (Figure 1C). Database searches with the NCBI genomic BLAST service also identified orthologs in several *B. pseudomallei* and *B. mallei* isolates. Seven *B. mallei* and twenty-nine *B. pseudomallei* strains for which sequences are available through the service were found to have the gene. Characteristics of these ORFs are listed in the Additional files 1 and 2. Overall, the proteins are 87-100% identical and differ primarily in the number and/or arrangement of SLST repeats, YadA stalk domains, and/or NSTA elements in their passenger domains. Based on these results, we conclude that BMA1027 orthologs are well-conserved gene products shared by *B. mallei* and *B. pseudomallei*. While preparing this article, Campos and colleagues published a report in which they functionally characterized autotransporter genes specified by the *B. pseudomallei* strain 1026b [51]. One of these molecules corresponds to the BMA1027 ortholog (locus tag # BP1026B_1575), which the authors designated *bpaC*. Henceforth, BMA1027 and orthologs will be referred to as BpaC.

### Expression and functional properties of the BpaC protein in *E. coli*

**Figure 2:**
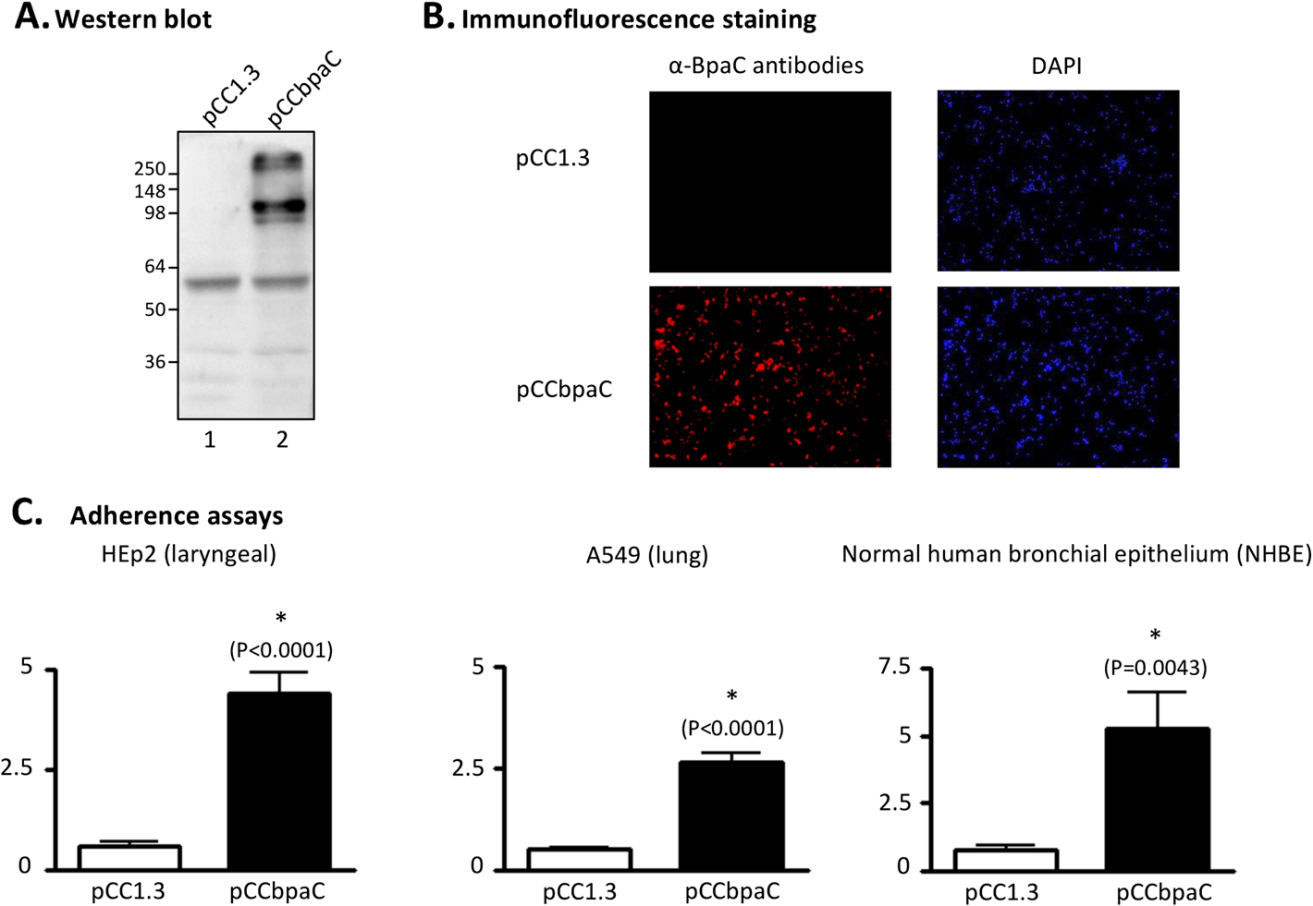
**Analysis of *E. coli *recombinant strains.** Panel **A**: Whole cell lysates were resolved by SDS-PAGE, transferred to PVDF membranes and analyzed by western blot with Abs against BpaC. Lane 1, *E. coli* (pCC1.3); lane 2, *E. coli* (pCCbpaC). MW markers are shown to the left in kilodaltons. Panel **B**: Non-permeabilized *E. coli* strains were fixed onto glass slides and fluorescently-labeled with DAPI (blue) and with α-BpaC Abs (red). Bacteria were visualized by microscopy using a Zeiss LSM 510 Meta confocal system. Representative microscopic fields are shown. Panel **C**: *E. coli* strains were incubated with epithelial cells for 3-hr. Cells were then washed to remove unbound bacteria, lysed, diluted and spread onto agar plates to enumerate bound bacteria. The results are expressed as the mean percentage (±standard error) of inoculated bacteria attached to epithelial cells. Asterisks indicate that the increased adherence of *E. coli* (pCCbpaC), compared to that of *E. coli* carrying the control plasmid pCC1.3, is statistically significant (*P* value shown in parentheses). Adherence assays were performed in duplicate on at least 4 independent occasions.

Because of sequence and structural similarities to known bacterial adhesins, we speculated that BpaC mediates adherence to epithelial cells. To test this hypothesis, the *bpaC* gene of *B. pseudomallei* DD503 was cloned and expressed in the *E. coli* strain EPI300. This organism does not adhere well to epithelial cells [8,53,55,62] and therefore provides a suitable heterologous genetic background to study the adherence properties of BpaC. To verify protein expression, whole cell lysates were prepared from *E. coli* EPI300 harboring the plasmid pCC1.3 (control) or pCCbpaC (specifies *B. pseudomallei* DD503 *bpaC*) and analyzed by western blot. Figure 2A shows that α-BpaC Abs (directed against aa 392–1098, part of surface-exposed passenger domain) react specifically with a band of 100-kDa in *E. coli* expressing the *bpaC* gene (lane 2), which is consistent with the predicted molecular mass of the gene product (Table 1). The Abs also specifically reacted with an antigen of high molecular weight (≥250 kDa), which likely corresponds to an oligomeric form of BpaC. Immunofluorescence-labeling of non-permeabilized *E. coli* cells was used to demonstrate that BpaC is displayed on the surface of recombinant bacteria. As shown in Figure 2B, *E. coli* carrying pCCbpaC is labeled by α-BpaC Abs while recombinant bacteria harboring the control plasmid pCC1.3 are not. Staining of nucleic acids with DAPI verified that equivalent numbers of bacteria were examined.

Quantitative adherence assays revealed that *E. coli* expressing BpaC binds to HEp-2 (laryngeal) and A549 (lung) human epithelial cells at levels 7- and 5-fold greater than bacteria carrying pCC1.3, respectively (Figure 2C). BpaC expression was also found to increase adherence by 7-fold to normal human bronchial epithelium (NHBE) cultured in an air-liquid interface system, which has been shown to represent an environment similar to the airway lumen in vivo [54,63,64]. These results demonstrate that BpaC mediates adherence to respiratory epithelial cells. *Burkholderia pseudomallei* and *B. mallei* are facultative intracellular bacteria that replicate within several eukaryotic cell types. Moreover, autotransporter adhesins frequently perform additional functions including invasion [1], intracellular motility [11], and survival inside host cells [10]. For these reasons, we examined the ability of *E. coli* expressing BpaC to invade epithelial cells and survive within murine macrophages. The results of these experiments indicated that BpaC does not substantially increase invasion of epithelial cells, phagocytosis of recombinant bacteria by J774A.1 murine macrophages, or survival inside these immune cells (data not shown).

### In vitro characterization of *B. mallei* and *B. pseudomallei* mutant strains

To study the functional properties of the *bpaC* gene product in *Burkholderia*, we constructed isogenic *bpaC* mutants of *B. pseudomallei* DD503 and *B. mallei* ATCC 23344. Whole cell lysates and sarkosyl-insoluble OM proteins were prepared from these strains and analyzed by western blot to verify lack of BpaC expression in the mutants. However, α-BpaC Abs did not react with protein preparations of parent or mutant strains (data not shown). Other methods such as immunoprecipitation and immunofluorescence-labeling also failed to detect BpaC expression. These results indicate that the *bpaC* gene is expressed at very low levels under the laboratory growth conditions we used to propagate the organisms.

**Figure 3:**
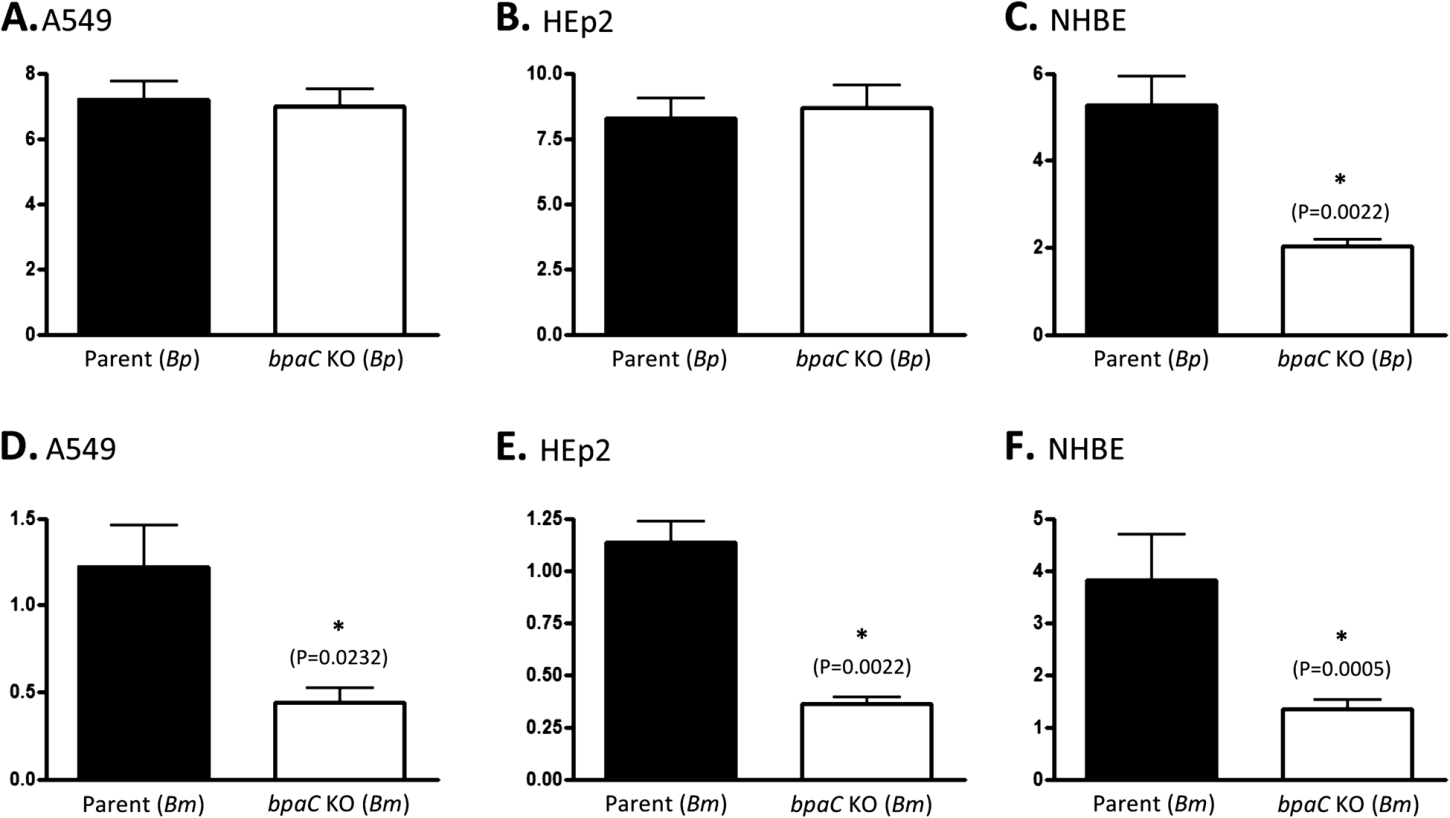
**Adherence of *B. mallei *and *B. pseudomallei *strains to human respiratory epithelial cells.** The effect of a *bpaC* mutation on the adherence of *B. pseudomallei* (*Bp*) DD503 and *B. mallei* (*Bm*) ATCC 23344 to monolayers of A549 (panels **A** and **D**) and HEp-2 (panels **B** and **E**) cells and cultures of NHBE (panels **C** and **F**) was measured in duplicate on at least 3 separate occasions. Strains were incubated with epithelial cells for 3-hr. Cells were then washed to remove unbound bacteria, lysed, diluted and spread onto agar plates to enumerate bound bacteria. The results are expressed as the mean percentage (±standard error) of inoculated bacteria adhering to epithelial cells. Asterisks indicate that the difference between the adherence of the *bpaC* KO mutant and that of the parent strain is statistically significant (*P* value shown in parentheses).

Because adherence assays with recombinant bacteria revealed that BpaC expression increases the binding of *E. coli* to NHBE cultures and monolayers of A549 and HEp-2 cells (Figure 2C), we compared the ability of *Burkholderia* parent and *bpaC* mutant strains to attach to these respiratory cells. Figure 3C shows that inactivation of the *bpaC* gene in *B. pseudomallei* DD503 affects adherence to NHBE cultures, reducing levels by 61%. The *B. pseudomallei* mutant bound to A549 and HEp-2 cells at wild-type levels. The *bpaC* mutation significantly impaired the ability of *B. mallei* ATCC 23344 to attach to A549 cells (66% reduction, Figure 3D), HEp-2 monolayers (72% reduction, Figure 3E), and NHBE cultures (66% reduction, Figure 3F). These results demonstrate that the *bpaC* gene product contributes to the adherence of *B. mallei* and *B. pseudomallei* to epithelial cells derived from the human respiratory tract.

As stated earlier, autotransporter adhesins often perform multiple biological functions including invasion [1] and survival within host cells [10]. In addition, *B. pseudomallei* and *B. mallei* are facultative intracellular pathogens that effectively replicate inside professional phagocytic cells. Therefore, we measured the ability of *Burholderia* mutant and parent strains to invade epithelial cells (A549 and HEp-2) and replicate within J774A.1 murine macrophages. The results of these experiments indicated that the *bpaC* mutation does not cause a defect in invasion of epithelial cells, phagocytosis of recombinant bacteria by J774A.1 murine macrophages, or growth inside these cells (data not shown). The *bpaC* mutants did not show defects in resistance to the bactericidal activity of normal human serum (data not shown), which is another biological function commonly associated with Oca autotransporters [2,3,19,65,66].

### Virulence of *B. mallei* and *B. pseudomallei* mutant strains and BpaC expression in vivo

To determine whether BpaC contributes to virulence, we calculated the median lethal dose (LD_50_) of *B. pseudomallei* and *B. mallei* mutant strains in a mouse model of aerosol infection. The model entails the use of a Microsprayer® to deliver bacteria directly into the murine lungs [67]. The device generates aerosol particles from the tip of a bent, 23-gauge nebulizing tube attached to a high-pressure stainless steel syringe that contains bacteria. BALB/c mice were anesthetized and placed in a custom-designed acrylic holder inside a Class II Biosafety cabinet. A modified pediatric otoscope equipped with a light source was then used to introduce the nebulizing tube portion of the Microsprayer® into the trachea of animals, and 50-μL of bacterial suspension was aerosolized into the lungs by pushing the plunger of the high-pressure syringe. Following infection, mice were observed daily for clinical signs of illness and morbidity. As shown in Table 2, the *bpaC* mutation did not have an impact on the LD_50_ values of *B. mallei* ATCC 23344 or *B. pseudomallei* DD503. Tissues (i.e. lungs, spleen, liver) from mice that survived the acute phase of infection did not show differences in bacterial loads (data not shown). Based on these results, we conclude that the *bpaC* mutation does not affect the virulence of *B. mallei* ATCC 23344 or *B. pseudomallei* DD503 via the aerosol route of infection.

**Table 2:** **Median lethal dose determination of *B. mallei *** **and *B. pseudomallei *** **WT and mutant strains**

**Organism**	**Strain**	**Inoculating dose (CFU)**	**Group size**	**% death**	**LD**_**50 **_**(CFU)**
*B. mallei*^*a*^	ATCC 23344 (WT)	9,100	5	100	
		5,550	5	100	
		910	9	78	346
		455	5	40	
		91	9	11	
*B. mallei*^*a*^	*bpaC* KO (mutant)	10,400	5	100	
		5,200	6	83	
		1,040	9	100	238
		520	5	40	
		104	9	22	
PBS (control)^*a*^		0	5	0	
*B. pseudomallei*^*b*^	DD503 (WT)	380,000	5	100	
		38,000	5	100	1,202
		3,800	5	100	
		380	5	0	
*B. pseudomallei*^*b*^	*bpaC* KO (mutant)	350,000	5	100	
		35,000	5	100	1,107
		3,500	5	100	
		350	5	0	
PBS (control)^*b*^		0	5	0	

^*a*^mice were monitored daily for clinical signs of illness/morbidity for 10 days post-inoculation.

^*b*^mice were monitored daily for clinical signs of illness/morbidity for 6 days post-inoculation.

**Figure 4:**
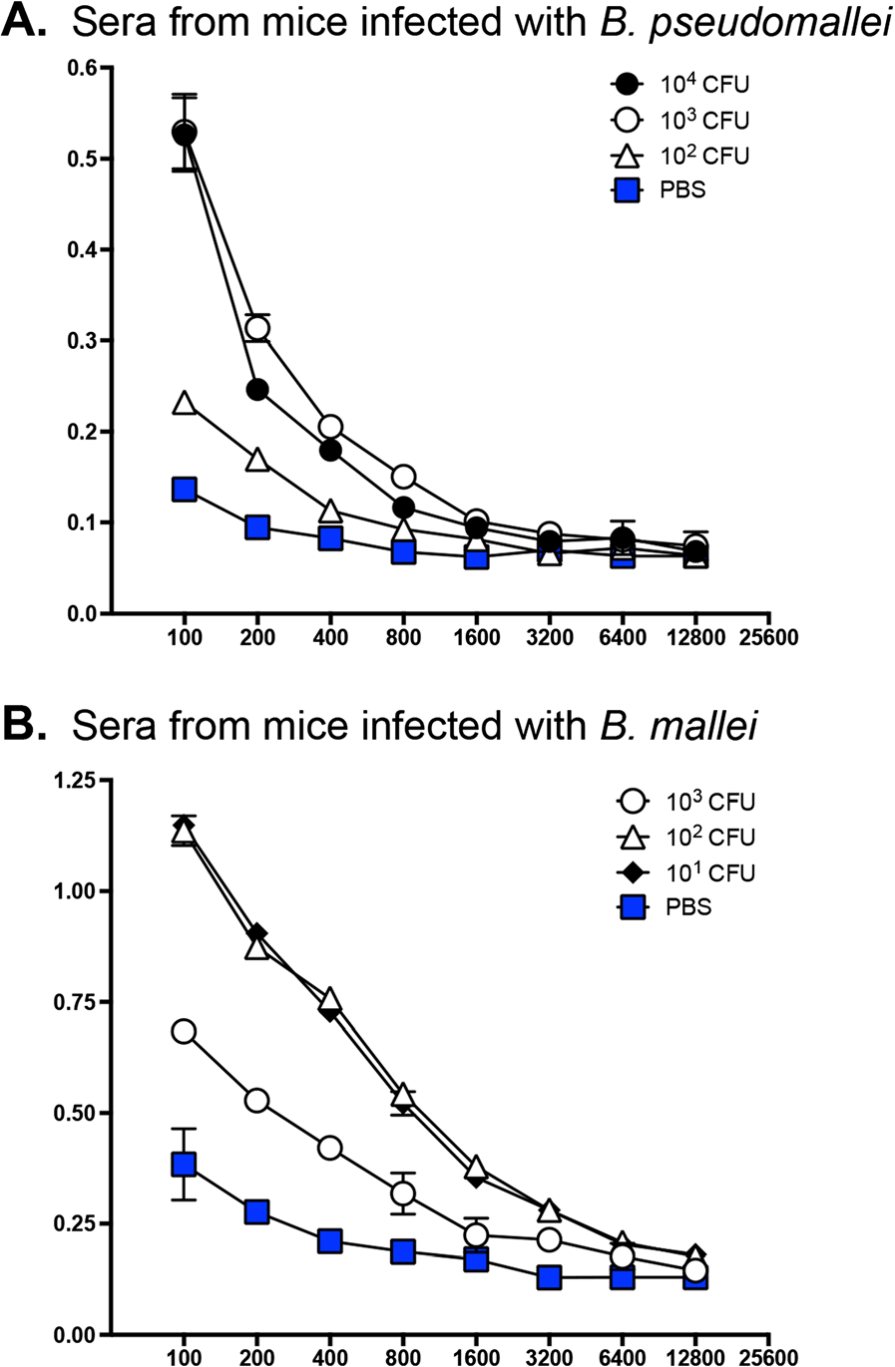
**ELISA with sera from mice that survived aerosol challenge with various doses of *B. pseudomallei *1026b and *B. mallei *ATCC 23344.** Serum samples were serially diluted and placed in duplicate wells of plates coated with purified His-tagged protein encompassing aa 392–1068 of *B. pseudomallei* 1026b BpaC. Goat α-mouse Abs conjugated to HRP were used as secondary Abs. The y-axis shows absorbance at a wavelength of 650 nm, which is indicative of antibody binding to antigens coating the plates. The x-axis represents two-fold dilutions of sera starting at 1:100 to 1:12,800. The results are expressed as the mean absorbance (±standard deviation). Closed circles show sera from mice inoculated with 10^4^*B. pseudomallei* bacteria (panel **A**). Open circles show sera from mice infected with 10^3^ organisms (panels **A** and **B**). Open triangles show sera from mice inoculated with 10^2^ bacteria (panels **A** and **B**). Closed diamonds show sera from mice infected with 10^1^ CFU of *B. mallei* ATCC 23344 (panel **B**). Blue squares represent sera from control mice that were inoculated with 50 μL of PBS using the Microsprayer (panels **A** and **B**). Of note, sera from mice that survived acute infection by *B. pseudomallei* and *B. mallei* are described elsewhere [67].

To gain insight into the immune response to BpaC during infection, we tested sera from mice that survived aerosol challenge with *B. mallei* ATCC 23344 and *B. pseudomallei* 1026b [67] by ELISA for the presence of Abs against the adhesin. Panels A and B in Figure 4 show that mice inoculated with doses as low as 10 CFU produced Abs against BpaC, which in turn demonstrates that this autotransporter is expressed by both *B. mallei* and *B. pseudomallei* during infection.

## Discussion

The genome of *B. mallei* ATCC 23344 has been reported to specify eight autotransporter gene products [49] and of these, only BoaA (adhesin, [55]) and BimA (intracellular motility protein, [11,68]) have been functionally characterized. Both are classified as oligomeric autotransporters because they possess a short C-terminal transporter module predicted to form 4 β-strands, which anchor the molecules on the bacterial surface. In the present study, we characterized a third *B. mallei* ATCC 2334 oligomeric autotransporter, BpaC (BMA1027). Comparative sequence analyses indicate that the gene product is conserved among *B. mallei* isolates (see Additional files 1 and 2) and resembles members of the Oca (oligomeric coiled-coil adhesin) sub-family of oligomeric autotransporter proteins [16,19-21]. Consistent with this, inactivation of *bpaC* in the genome of *B. mallei* ATCC 23344 reduces adherence to monolayers of A549 (lung) and HEp-2 (larynx) cells grown in submerged cultures (Figure 3D and E, respectively). Though these cells are relevant to aerosol infection by *B. mallei*, they lack key features of the airway mucosa such as cilia and mucociliary activity. Ciliated cells contribute to preventing colonization of the respiratory tract by pathogenic agents by moving secretions (and trapped organisms) toward the laryngopharynx for expectoration or swallowing to the stomach (where the acidic pH neutralizes organisms). For these reasons, we measured the adherence of the *B. mallei bpaC* mutant to cultures of NHBE grown in an air-liquid interface system. These cultures mimic the structure and function of the airway mucosa as they form a pseudostratified epithelium with tight junctions, contain ciliated and mucus-producing goblet cells, and display mucociliary activity [63,64]. Quantitative assays using this system revealed that adherence of the *bpaC* mutant was reduced by 66% (Figure 3F).

Orthologs of BpaC were identified in 29 *B. pseudomallei* isolates (see Additional files 1 and 2). The genome of some of these strains has not been completed, resulting in the passenger domain and transporter module of BpaC seemingly specified by two different ORFs (*e. g.* B7210, 112, BPC006, 354e). Inactivation of *bpaC* in the genome of the *B. pseudomallei* strain DD503 caused a 2.6-fold reduction in adherence to NHBE cultures (Figure 3C), which is consistent with the phenotype of the *B. mallei bpaC* mutant (Figure 3F). However, the *bpaC* mutation did not affect adherence of *B. pseudomallei* to A549 or HEp-2 cells (Figure 3A and B, respectively). One possible explanation for this lack of effect is that other adhesins expressed by the *B. pseudomallei* DD503 *bpaC* mutant provide a high background of adherence to A549 and HEp-2 monolayers. For instance, BoaA and BoaB have been shown to mediate binding of *B. pseudomallei* DD503 to HEp-2 and A549 cells [55]. Moreover, it was recently demonstrated that the *B. pseudomallei* gene products BpaA, BpaB, BpaD, BpaE and BpaF all play a role in adherence to A549 cells [51]. The genes encoding these molecules are present in the genome of strain DD503.

While preparing this article, Campos and colleagues published a study in which they demonstrate that BpaC is an adhesin for A549 cells [51]. The authors reported that a mutation in the *bpaC* gene of *B. pseudomallei* strain 340 causes an ~ 10-fold reduction in adherence. These results are in contrast with our data showing that a *B. pseudomallei* DD503 *bpaC* mutant binds to A549 cells at wild-type levels (Figure 3A). One possible explanation for this phenotypic difference is that we performed adherence assays using plate-grown bacteria, and infected A549 cells for 3 hours before washing off unbound *B. pseudomallei* and measuring cell-binding. Campos et al. used overnight broth cultures to inoculate A549 cells and infected monolayers for only 2 hours. The method used to construct mutants might have impacted the experimental outcome of adherence assays as well. In the present study, an internal portion of the *bpaC* ORF was replaced with a zeocin resistance marker and this mutation was introduced in the genome of *B. pseudomallei* DD503 via allelic exchange. In contrast, the *bpaC* gene of *B. pseudomallei* strain 340 was disrupted via co-integration of a large plasmid (~9-kb) in the genome [51]. It is unlikely that the strain background in which the mutations were constructed has any effect on the phenotype of *bpaC* mutants since strains DD503 [61] and 340 [69] are both antibiotic-sensitive derivatives of the same clinical isolate, *B. pseudomallei* 1026b. Despite these differences, our data constitute independent proof of the role of BpaC as an adhesin. These results are substantiated by showing that expression of BpaC on the surface of recombinant *E. coli* bacteria increases adherence to NHBE, A549 and HEp-2 cells (Figure 2).

Given the phenotype of mutants in assays with NHBE cultures and that adherence is a key step in pathogenesis by most infectious agents, we expected the *bpaC* mutation to reduce the virulence of *B. mallei* and/or *B. pseudomallei* in a mouse model of aerosol infection. However, the results of our animal experiments indicate that the mutants are as virulent as wild-type strains (Table 2). Presumably, other adhesins expressed by the *bpaC* mutants provide sufficient adherence to the murine airway mucosa to allow colonization at wild-type levels and for the normal course of disease to ensue. It is unlikely that the lack of phenotype we observed in vivo is due to non-expression of BpaC. Though we discovered that *B. pseudomallei* DD503 and *B. mallei* ATCC 23344 do not produce detectable amounts of BpaC under routine laboratory growth conditions, ELISA with sera from mice that survived acute aerosol infection with the agents show that animals produce Abs against the protein (Figure 4A and B). Moreover, sera from horses with experimental glanders have been shown to contain high antibody titers against BpaC [70]. These results are particularly significant as horses are the natural host and reservoir for *B. mallei* and arguably the most relevant surrogate to study glanders. Together, these data demonstrate that BpaC is expressed in vivo and elicits the production of Abs during infection.

The infection model we used to examine the effect of the *bpaC* mutation might have impacted the outcome of virulence experiments. This hypothesis is supported by the Campos et al. study in which they show that the *bpaC* mutation reduces the ability of *B. pseudomallei* strain 340 to disseminate and/or survive in the liver [51]. In these experiments, BALB/c mice were infected intranasally with 500 CFU of the agent and bacterial loads in tissues were determined 48 hours post-infection. In contrast, we inoculated BALB/c mice intratracheally using a Microsprayer®, which nebulizes bacteria directly into the lungs, infected animals with doses ranging from 10^2^ to 10^5^ CFU, and determined bacterial burden in survivors 6–10 days post-infection (Table 2). It is also known that the choice of bacterial strains [71], inoculation route [72], and animal background [73] can significantly affect the course of disease by *B. pseudomallei* and *B. mallei*. For example, the LD_50_ value of the same *B. pseudomallei* isolate has been shown to differ by several orders of magnitude in C57BL/6 mice and BALB/c mice [74]. Therefore, a complete understanding of the role of BpaC in pathogenesis may require the use of multiple infection models.

## Conclusion

This study demonstrates that BpaC is an autotransporter protein, which mediates adherence of *B. mallei* and *B. pseudomallei* to host cells that are relevant to pathogenesis by the organisms. We show that BpaC is conserved among isolates of both *Burkholderia* species, is expressed in vivo, and elicits production of Abs during infection. Hence, BpaC displays many properties of an important virulence factor and potential target for developing countermeasures. Though our animal experiments indicate that a mutation in *bpaC* does not impact the virulence of *B. mallei* or *B. pseudomallei*, adherence to host surfaces is a key early step in pathogenesis by most infectious agents. To accomplish this, pathogenic organisms typically express multiple adhesins to ascertain host colonization. It is likely that disruption of multiple genes specifying adherence factors, including *bpaC*, will result in decreased virulence and clarify the role of the autotransporter in the pathogenesis of *B. mallei* and *B. pseudomallei*. Continued investigation of BpaC will yield important information regarding the complex biology and virulence of these organisms, and may contribute to development of comprehensive countermeasures targeting autotransporters and their roles in pathogenesis.

## Methods

### Strains, plasmids, tissue culture cell lines and growth conditions

The strains and plasmids used in this study are listed in Table 3. For construction of the *B. pseudomallei bpaC* mutant, Low Salt Luria Bertani (LSLB) agar (Teknova) supplemented with antibiotics was utilized as selective medium. For all other experiments, *B. pseudomallei* was cultured on Trypticase Soy Agar (BD) at 37°C. Brucella Agar (BD) supplemented with 5% glycerol was used to grow *Burkholderia mallei* at 37°C. Where indicated, antibiotics were added to the culture media at the following concentrations: 7.5 μg/mL (for *B. mallei*) and 100 μg/mL (for *B. pseudomallei*) Polymixin B (MP Biomedicals), 7.5 μg/mL (for *B. mallei*) and 50 μg/mL (for *B. pseudomallei*) kanamycin (MP Biomedicals), 7.5 μg/mL (for *B. mallei*) and 100 μg/mL (for *B. pseudomallei*) zeocin™ (Life Technologies™). Plate-grown bacteria (40-hr for *B. mallei*, 20-hr for *B. pseudomallei*) were used for all experiments. For conjugative transfer of plasmids from *E. coli* to *Burkholderia*, MgSO_4_ was added to culture media at a final concentration of 10 mM.

**Table 3:** Strains and plasmids

**Strain/plasmid**	**Description**	**Reference**
*B. pseudomallei*		
DD503	Parental strain; polymixin B resistant, zeocin sensitive, kanamycin sensitive (derived from clinical isolate 1026b)	[61]
*bpaC* KO	Isogenic *bpaC* mutant strain of DD503; polymixin B resistant, zeocin resistant, kanamycin sensitive	This study
*B. mallei*		
ATCC 23344	Wild-type strain; polymixin B resistant, zeocin sensitive, kanamycin sensitive	[75]
*bpaC* KO	Isogenic *bpaC* mutant strain of ATCC 23344; polymixin B resistant, zeocin resistant, kanamycin sensitive	This study
*E. coli*		
EPI300	Cloning strain	epicentre® Illumina®
TUNER™	Expression strain for purification of His-tagged BpaC	EMD Millipore
S17	Strain used for conjugational transfer of pKASbpaC.zeo to *B. pseudomallei* and *B. mallei*	[76]
pCC1™	Cloning vector, chloramphenicol resistant	epicentre® Illumina®
pCCbpaC	pCC1 containing the *B. pseudomallei* DD503 *bpaC* gene, chloramphenicol resistant	This study
pCCbpaC.zeo	pCCbpaC in which a zeocin resistance cassette was introduced near the middle of the *bpaC* ORF; chloramphenicol and zeocin resistant	This study
pCC1.3	pCC1-based plasmid control, does not confer adherence to human epithelial cells; chloramphenicol resistant	[77]
pKAS46	Mobilizable suicide plasmid; kanamycin resistant	[78]
pKASbpaC.zeo	pKAS46 containing the insert from pCCbpaC.zeo	This study
pEM7ZEO	Source of the zeocin resistance marker	Life Technologies™
pELHisBPSL1631-BMA1027	Plasmid expressing aa 392–1068 of *B. pseudomallei* 1026b BpaC fused to six N-terminal histidine residues, introduced in *E. coli* TUNER and used to purify His-tagged BpaC protein for antibody production and ELISA experiments; chloramphenicol resistant.	[67]

*Escherichia coli* was cultured at 37°C using LSLB supplemented with 15 μg/mL chloramphenicol, 50 μg/mL kanamycin, or 50 μg/mL zeocin, where indicated. For conjugation experiments, LSLB was supplemented with 10 mM MgSO_4_. For assays with *E. coli* clones carrying pCC1-based plasmids, the CopyControl™ Induction Solution (epicentre® Illumina®) was added to LSLB as previously reported [9].

The cell lines HEp-2 (human laryngeal epithelium; ATCC CCL-23), A549 (type II alveolar epithelium; ATCC CCL85) and J774A.1 (murine macrophages; ATCC TIB-67) were cultured as outlined by others [5,55]. Normal human bronchial epithelium (NHBE; LONZA) was expanded, cryopreserved and cultured in an air-liquid interface system as previously described [54,63,64]. The apical surface of the NHBE was exposed to air for a minimum of 3 weeks prior to use in adherence assays to ascertain proper cellular differentiation and the development of functional cilia.

### Recombinant DNA methodology

Standard molecular biology techniques were performed as described elsewhere [79]. Genomic DNA was purified from *Burkholderia* using the Easy-DNA™ Kit (Life Technologies™). Plasmid DNA was isolated with the QIAprep Spin Miniprep kit (QIAGEN). The Platinum® *Pfx* DNA Polymerase (Life Technologies™) was used to amplify the 3.8-kb *bpaC* gene of *B. pseudomallei* DD503 with primers P1 (5’-ATA CCC AAA TCG GCG TTC TCT GGT-3′) and P2 (5′-TGC GCG AAT CAA TCG AGA TAC CCA-3′) and the PCR product was used as a template in sequencing reactions. The amplicon was also cloned in the vector pCC1™ using the CopyControl™ PCR cloning kit (epicentre® Illumina®), producing the plasmid pCCbpaC (Table 3). The latter was sequenced to determine that PCR did not introduce mutations resulting in aa substitutions in the *bpaC* gene product.

### Construction of isogenic mutant strains of *B. mallei* and *B. pseudomallei*

The plasmid pCCbpaC was digested with the enzyme *Nsi*I (New England BioLabs®, Inc.) to remove a 0.6-kb fragment internal to the *bpaC* ORF, treated with the End-It™ DNA End Repair Kit (epicentre® Illumina®), and ligated with a 0.4-kb zeocin resistance cassette to yield the construct pCCbpaC.zeo. This plasmid was restricted with *Bam*HI (New England BioLabs®, Inc.) and a 3.4-kb fragment corresponding to the *bpaC* ORF disrupted by the insertion of the zeocin resistance cassette was excised from an agarose gel, purified with the High Pure PCR Product Purification kit (Roche Applied Science), and treated with the End-It™ DNA End Repair Kit. This blunt DNA fragment was then cloned in the suicide vector pKAS46. The resulting plasmid, designated pKASbpaC.zeo, was introduced in the *E. coli* strain S17 by electroporation, and subsequently transferred into *B. mallei* ATCC 23344 or *B. pseudomallei* DD503 by conjugation, as previously reported [55,80]. Upon conjugation, *Burkholderia* colonies were selected for resistance to zeocin. These putative mutants were then screened by PCR using Platinum® *Pfx* DNA Polymerase with primers P1 and P2. The primers yielded a PCR product of 3.8-kb in the parent strains and a smaller amplicon of 3.6-kb in *bpaC* mutants. The PCR products from mutant strains were sequenced to verify proper allelic exchange and successful disruption of *bpaC*.

### Nucleotide sequence and bioinformatic analyses

PCR products and plasmids were sequenced at the University of Michigan Sequencing Core (http://seqcore.brcf.med.umich.edu). Chromatograms were assembled using the Sequencher® 5 software (Gene Codes Corporation). Sequence analyses were performed using Vector NTI (Life Technologies™) and the various online tools available through the EsPASy Bioinformatics Resource Portal (http://www.expasy.org). Signal sequence cleavage sites were determined using the SignalP 4.1 server (http://www.cbs.dtu.dk/services/SignalP). The *B. mallei* ATCC 23344 *bpaC* gene product (locus tag # BMA1027) was identified by searching the genome of the organism for the presence of a YadA anchor domain (Pfam database number PF3895.10) through the NCBI genomic BLAST service using the blastp program (http://www.ncbi.nlm.nih.gov/sutils/genom_table.cgi). The other *bpaC* gene products described in this study were identified using the predicted aa sequence of the *B. mallei* ATCC 23344 BpaC protein to search the genomes of the *B. mallei* and *B. pseudomallei* strains available through the NCBI genomic BLAST service utilizing the tblastn and blastp programs. Structural features of the BpaC proteins (helical regions, hydrophobic β-strands) were identified with the PSIPRED Protein Sequence Analysis Workbench service (http://bioinf.cs.ucl.ac.uk/psipred/).

### Experiments with epithelial cells and J774 murine macrophages

Adherence, invasion, and intracellular survival assays were performed as previously reported by our laboratory [53-55]. Cells were inoculated with bacteria at a multiplicity of infection (MOI) of 100. Duplicate assays were performed on at least 3 occasions. Statistical analyses were performed using the Mann–Whitney test (GraphPad Prism® 6 software) and *P* values < 0.05 are reported as statistically significant.

### Bactericidal assays

The method used to examine the effect of *bpaC* mutations on the ability of *Burkholderia* to resist the bactericidal activity of complement is outlined elsewhere [9,77,81]. We used final concentrations of 50% and 25% serum in assays with *B. pseudomallei* and *B. mallei*, respectively.

### Protein preparations, western blot, purification of recombinant BpaC protein, and antibody production

Sarkosyl-insoluble OM protein preparations were obtained as described by Carlone et al. [82]. The methods used to prepare whole cell lysates and perform western blot experiments are described elsewhere [8,53,54,57,83,84]. His-tagged recombinant BpaC was obtained from cultures of *E. coli* TUNER carrying the plasmid pELHisBPSL1631-BMA1027, as previously outlined by our laboratory [67]. To obtain polyclonal Abs directed against BpaC, the purified His-tagged protein was emulsified in Freund’s adjuvants (SIGMA-ALDRICH®) and used to immunize female BALB/c mice as reported by Lafontaine and colleagues [85].

### Immunofluorescence labeling of *E. coli* and microscopy

Expression of BpaC on the surface of *E. coli* recombinant bacteria was visualized by immunofluorescence microscopy as outlined by Balder et al. [55]. Briefly, paraformaldehyde-fixed *E. coli* cells were spotted onto glass slides. These bacteria were probed with α-BpaC polyclonal Abs, followed by incubation with a goat α-mouse antibody labeled with Alexa Fluor 546® (Life Technologies™) and the nucleic acid dye DAPI (Life Technologies™). Slides were examined by microscopy using a Zeiss LSM 510 Meta confocal system.

### ELISA

Duplicate wells of Immulon™ 2HB plates (Thermo Scientific Nunc) were coated overnight at 4C° with 1 μg of His-tagged BpaC. Excess unbound antigen was removed by washing the wells with PBS + 0.05% Tween 20 (PBST), and the wells were then blocked with PBS + 0.05% containing 3% dry milk (blocking buffer) for 1 hour at room temperature. After washing with PBST, the wells were probed overnight at 4°C with sera from mice that survived acute aerosol infection with *B. mallei* ATCC 23344 and *B. pseudomallei* 1026b [67] diluted in blocking buffer. After this incubation, the wells were washed with PBST and incubated overnight with a goat α-mouse antibody conjugated to Horse Radish Peroxidase (SouthernBiotech) diluted in blocking buffer. After washing off the excess secondary antibody with PBST, 100 μL of the SureBlue™ TMB Microwell Peroxidase Substrate (KPL) was added to the wells. Color development, which is indicative of Abs binding to BpaC, was measured spectrophotometrically by determining the absorbance of well contents at a wavelength of 650 nm.

### Animal experiments

Female BALB/c mice (6–8 weeks of age) were purchased from Frederick National Laboratory for Cancer Research. The animals were anesthetized by injecting a dose of 250 mg/kg of 2,2,2 tribromoethanol (SIGMA-ALDRICH®) intraperitoneally. Once anesthetized, mice were inoculated intratracheally with 50 μL of bacterial suspensions using a Microsprayer® model I-1C (PennCentury™) as previously reported by our laboratory [67]. Infected animals were monitored twice daily. Humane end-points were strictly observed. Mice exhibiting signs of moderate to severe discomfort were euthanized. This was accomplished by anesthetizing the animals with 2,2,2 tribromoethanol followed by cervical dislocation, in accordance with the AVMA Guidelines on euthanasia. Food and water were provided ad libitum. Analgesics were not used as they may have affected the experimental outcomes of the studies. Survival data were analyzed using the Kaplan-Meier method and the LD_50_ values were calculated according to Reed and Muench [86].

### Compliance and animal research ethic statements

All experiments with live *B. pseudomallei* and *B. mallei* were performed inside a Class II Biosafety Cabinet in a BSL3 laboratory and in compliance with the rules and regulations of the U.S. Federal Select Agent Program. The experiments were approved by the University of Georgia’s Institutional Biosafety Committee (IBC).

Animal experiments were carried out in strict accordance with the recommendations in the Guide for the Care and Use of Laboratory Animals of the National Institutes of Health. The experiments were approved by the University of Georgia’s Institutional Animal Care and Use Committee (IACUC). All efforts were made to minimize animal suffering.

## Abbreviations

aa: Amino acid; OM: Outer membrane; Oca: Oligomeric coiled-coil adhesin; Bp: *Burkholderia pseudomallei*; Bm: *Burkholderia mallei*; KO: Knock-out; Abs: Antibodies; LD50: Median lethal dose; LSLB: Low salt luria bertani; CFU: Colony forming units; ORF: Open reading frame; NHBE: Normal human bronchial epithelium.

## Competing interests

ERL, RB, FM and RJH do not have financial or non-financial competing interests. In the past five years, the authors have not received reimbursements, fees, funding, or salary from an organization that may in any way gain or lose financially from the publication of this manuscript, either now or in the future. Such an organization is not financing this manuscript. The authors do not hold stocks or shares in an organization that may in any way gain or lose financially from the publication of this manuscript, either now or in the future. The authors do not hold and are not currently applying for any patents relating to the content of the manuscript. The authors have not received reimbursements, fees, funding, or salary from an organization that holds or has applied for patents relating to the content of the manuscript. The authors do not have non-financial competing interests (political, personal, religious, ideological, academic, intellectual, commercial or any other) to declare in relation to this manuscript.

## Authors’ contributions

Conceived and designed the experiments: ERL and RJH. Performed the experiments: ERL, FM, RB. Analyzed the data: ERL, RB, RJH. Wrote the manuscript: ERL and RJH. All authors read and approve the final manuscript.

## Supplementary Material

Additional file 1 **Comparison of the structural features specified by *B. pseudomallei *** **and *B. mallei bpaC *** **gene products.** Click here for file

Additional file 2 **Characteristics**^**a **^**of BMA1027 orthologous genes and their encoded products.** Click here for file
