# Supplementary figures and images for: Characterization of an autotransporter adhesin protein shared by Burkholderia mallei and Burkholderia pseudomallei
Source: BMC Microbiol. 2014 Apr 14;14:92. doi: 10.1186/1471-2180-14-92 (PMC4021183; doi:10.1186/1471-2180-14-92)

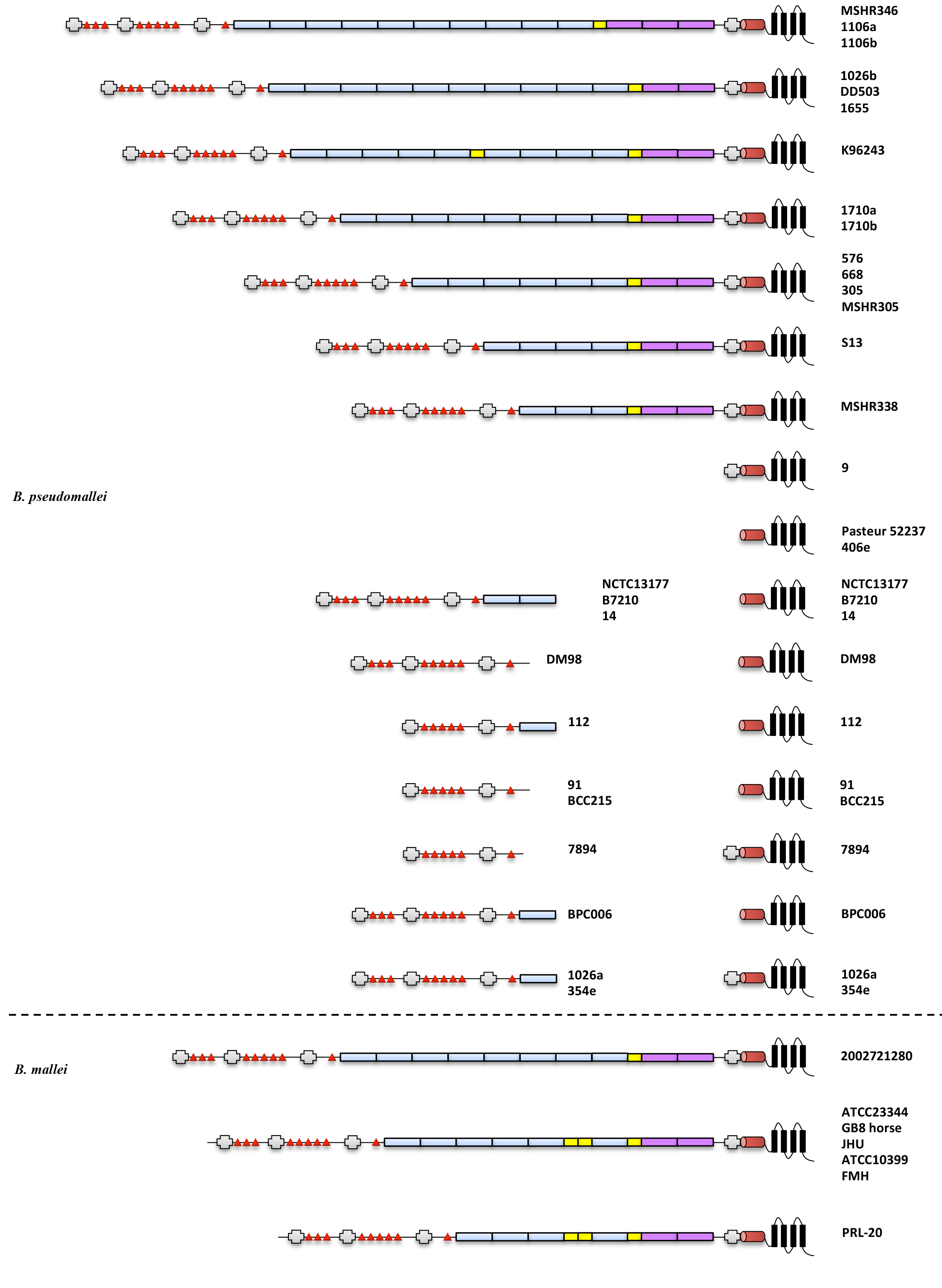

Supplement: Additional file 1 — Comparison of the structural features specified by B. pseudomallei and B. mallei bpaC gene products. [file 1471-2180-14-92-S1.tiff]
