# Supplementary material for: Characterization of an autotransporter adhesin protein shared by Burkholderia mallei and Burkholderia pseudomallei
Source: BMC Microbiol. 2014 Apr 14;14:92. doi: 10.1186/1471-2180-14-92 (PMC4021183; doi:10.1186/1471-2180-14-92)
Supplement: Additional file 2 — Characteristics a of BMA1027 orthologous genes and their encoded products. [file 1471-2180-14-92-S2.doc]

**Additional file 2: Characteristics^a^ of BMA1027 orthologous genes and their encoded products**

| **Strain^b^** | **Locus tag** | **Predicted protein (aa)** | **MW (kDa)** | **Potential signal sequence cleavage site^c^** | **Comments** |
| --- | --- | --- | --- | --- | --- |
| ***B. pseudomallei*** |  |  |  |  |  |
| 1106A | BURPS1106A_2083 | 1,186 | 109.2 | ASA^14▼^G, AMA^46▼^A |  |
| 1106B | BURPS1106B_A1319 | 1,186 | 109.2 | ASA^14▼^G, AMA^46▼^A |  |
| MSHR346 | GBP346_A2154 | 1,185 | 109.3 | ASA^14▼^G, AMA^46▼^A |  |
| 1026b/DD503* | BP1026B_I1575 | 1,152 | 107.4 | ASA^37▼^G, AMA^69▼^A |  |
| 1655 | BURPS1655_A1682 | 1,152 | 107.3 | ASA^37▼^G, AMA^69▼^A |  |
| K96243 | BPSL1631 | 1,124 | 104.8 | ASA^37▼^G, AMA^69▼^A |  |
| 1710A | BURPS1710A_2576 | 1,068 | 99.8 | ASA^37▼^G, AMA^69▼^A |  |
| 1710B | BURPS1710B_2229 | 1,068 | 99.8 | ASA^37▼^G, AMA^69▼^A |  |
| 576 | BUC_2000 | 985 | 92.2 | ASA^37▼^G, AMA^69▼^A |  |
| MSHR305 | BDL_174 | 984 | 92.3 | ASA^37▼^G, AMA^69▼^A |  |
| 668 | BURPS668_2027 | 962 | 89.4 | ASA^14▼^G, AMA^46▼^A |  |
| 305 | BURPS305_6685 | 961 | 89.6 | ASA^14▼^G, AMA^46▼^A |  |
| S13 | BURPSS13_PO706 | 900 | 84.7 | ASA^37▼^G, AMA^69▼^A |  |
| MSHR338 | M218_09845 | 753 | 70.4 | LTA^14▼^D |  |
| 1258b | BP1258B_1342 | 315 | 28.9 | None detected | Specifies truncated passenger domain, linker and transporter module. |
| 1026a | BP1026A_6285 | 462 | 44.8 | ASA^37▼^G, AMA^69▼^A | Specifies truncated passenger domain only. |
| 1026a | BP1026A_0706 | 286 | 26.4 | None detected | Specifies linker and transporter module. |
| 354e | BP1026A_6285 | 462 | 44.8 | ASA^37▼^G, AMA^69▼^A | Specifies truncated passenger domain only. |
| 354e | BP354E_5332 | 318 | 29.3 | None detected | Specifies linker and transporter module. |
| NCTC13177 | BpseN_010100009742 | 537 | 51.4 | ASA^37▼^G, AMA^69▼^A | Specifies passenger domain only. |
| NCTC13177 | BpseN_010100009762 | 98 | 10.2 | None detected | Specifies linker and transporter module. |
| B7210 | BpseB_010100009783 | 519 | 49.7 | ASA^37▼^G, AMA^69▼^A | Specifies passenger domain only. |
| B7210 | BpseB_010100009798 | 98 | 10.2 | None detected | Specifies linker and transporter module. |
| 14 | Bpse14_010100010165 | 436 | 41.2 | LTA^14▼^D | Specifies truncated passenger domain only. |
| 14 | Bpse14_010100010180 | 98 | 10.2 | None detected | Specifies linker and transporter module. |
| 7894 | Bpse7_010100010166 | 251 | 23.8 | None detected | Specifies truncated passenger domain only. |
| 7894 | Bpse7_010100010171 | 221 | 20.9 | AAA^20▼^G | Specifies linker and transporter module. |
| DM98 | BpseD_010100010387 | 347 | 33.3 | LTA^14▼^D | Specifies truncated passenger domain only. |
| DM98 | BpseD_010100010402 | 98 | 10.2 | None detected | Specifies linker and transporter module. |
| 112 | Bpse112_010100009840 | 274 | 25.8 | None detected | Specifies truncated passenger domain only. |
| 112 | Bpse112_010100009855 | 87 | 9 | ATA^24▼^L | Specifies linker and transporter module. |
| BPC006 | BPC006_I2129 | 465 | 44.3 | ASA^14▼^G, AMA^46▼^A | Specifies truncated passenger domain only. |
| BPC006 | BPC006_I2127 | 87 | 9 | ATA^24▼^L | Specifies linker and transporter module. |
| 91 | Bpse9_010100010769 | 265 | 25.1 | None detected | Specifies truncated passenger domain only. |
| 91 | Bpse9_010100010779 | 87 | 9 | ATA^24▼^L | Specifies linker and transporter module. |
| BCC215 | BpseBC_010100009715 | 246 | 23.3 | None detected | Specifies truncated passenger domain only. |
| BCC215 | BpseBC_010100009730 | 87 | 9 | ATA^24▼^L | Specifies linker and transporter module. |
| 9 | Bpseu9_010100010125 | 227 | 21.5 | AAA^26▼^G | Specifies linker and transporter module. Does not contain passenger domain. |
| Pasteur 52237 | BURPSPAST_AA0768 | 98 | 10.2 | None detected | Specifies linker and transporter module. Does not contain passenger domain. |
| 406E | BURPS406E_H0892 | 87 | 9 | ATA^24▼^L | Specifies linker and transporter module. Does not contain passenger domain. |
| ***B. mallei*** |  |  |  |  |  |
| 2002721280 | BMA721280_A0398 | 1,045 | 97 | ASA^14▼^G, AMA^46▼^A |  |
| ATCC23344 | BMA1027 | 1,012 | 94.7 | ASA^37▼^G, AMA^69▼^A |  |
| GB8 | BMAGB8_1092 | 1,012 | 94.7 | ASA^37▼^G, AMA^69▼^A |  |
| JHU | BMAJHU_CO589 | 1,012 | 94.7 | ASA^37▼^G, AMA^69▼^A |  |
| ATCC10399 | BMA10399_E0973 | 989 | 92.1 | ASA^14▼^G, AMA^46▼^A |  |
| FMH | BMAFMH_C0552 | 989 | 92 | ASA^14▼^G, AMA^46▼^A |  |
| PRL-20 | BMAPRL20_A1011 | 928 | 87.2 | ASA^37▼^G, AMA^69▼^A |  |

^a^ Sequence analyses were performed using Vector NTI (Life Technologies^TM^) and online tools available through the ExPASy Bioinformatics Resources Portal.

^b^ *B. pseudomallei* and *B. mallei* isolates for which genomic sequences are available through the NCBI genomic BLAST service.

^c^ The putative signal sequence cleavage sites were determined using the SignalP 4.1 server.

^*^ The *B. pseudomallei* strain DD503 is a derivative of isolate 1026b in which the AmrAB-OprA antibiotic efflux pump has been deleted to facilitate mutant construction . The BMA1027 orthologs of strains DD503 and 1026b are identical (confirmed by nucleotide sequence analysis, data not shown).
